# Near-Infrared Hyperspectral Imaging Combined with Deep Learning to Identify Cotton Seed Varieties

**DOI:** 10.3390/molecules24183268

**Published:** 2019-09-07

**Authors:** Susu Zhu, Lei Zhou, Pan Gao, Yidan Bao, Yong He, Lei Feng

**Affiliations:** 1 College of Biosystems Engineering and Food Science, Zhejiang University, Hangzhou 310058, China; 2 Key Laboratory of Spectroscopy Sensing, Ministry of Agriculture and Rural Affairs, Hangzhou 310058, China; 3 College of Information Science and Technology, Shihezi University, Shihezi 832000, China

**Keywords:** near-infrared hyperspectral imaging, cotton seed, convolution neural network, residual network, classifier

## Abstract

Cotton seed purity is a critical factor influencing the cotton yield. In this study, near-infrared hyperspectral imaging was used to identify seven varieties of cotton seeds. Score images formed by pixel-wise principal component analysis (PCA) showed that there were differences among different varieties of cotton seeds. Effective wavelengths were selected according to PCA loadings. A self-design convolution neural network (CNN) and a Residual Network (ResNet) were used to establish classification models. Partial least squares discriminant analysis (PLS-DA), logistic regression (LR) and support vector machine (SVM) were used as direct classifiers based on full spectra and effective wavelengths for comparison. Furthermore, PLS-DA, LR and SVM models were used for cotton seeds classification based on deep features extracted by self-design CNN and ResNet models. LR and PLS-DA models using deep features as input performed slightly better than those using full spectra and effective wavelengths directly. Self-design CNN based models performed slightly better than ResNet based models. Classification models using full spectra performed better than those using effective wavelengths, with classification accuracy of calibration, validation and prediction sets all over 80% for most models. The overall results illustrated that near-infrared hyperspectral imaging with deep learning was feasible to identify cotton seed varieties.

## 1. Introduction

Cotton (*Gossypium* spp.) is one of the most widely cultivated economic crops in the world, especially in China. What’s more, cotton is the main source of natural fiber, vegetable oil and high protein meals for humans and livestock [1]. Cotton seeds of different varieties show different characteristics relating to vigor, germination, yield, quality and stress resistance, etc. Thus, cotton seed purity is an important factor for cotton yield, which is also closely related to the income of farmers.

Seed variety identification is an important issue for crop planting. Gene based techniques can be used to identify seed varieties accurately [2,3]. However, the sample preparation is complex for these methods, and workers with expert operation skill are also indispensable. Moreover, the inefficiency and high costs also make gene based techniques unable to adapt to the needs of market. This situation leads to the urgent demand of developing rapid and non-destructive techniques.

Machine vision is a rapid and non-destructive technique which could be used to identify seed varieties, with the advantages of simple sample preparation, batch detection and good adaptability to different application scenes. Moreover, it is easy to obtain the required images. Extraction of features, such as shape, texture, color and etc., is the key factor for seed variety identification. Studies have proved the feasibility to identify seed varieties using machine vision [4,5].

Near-infrared spectroscopy is another rapid and non-destructive technique which could be applied to identify seed varieties [6]. Near-infrared spectroscopy acquires near-infrared spectra which relate to the chemical compositions of seeds. Near-infrared spectroscopy also has the advantage of minimum sample preparation and batch detection. Studies have proved the possibility to discriminant seed varieties using near-infrared spectroscopy [7,8,9].

Machine vision mainly focuses on spatial information but lacks composition information, while near-infrared spectroscopy is on the contrary. Hyperspectral imaging, a technique integrating both machine vision and near-infrared spectroscopy, can acquire spectral and spatial information simultaneously. This advantage of hyperspectral imaging enables its wide application in various fields [10,11,12,13].

Hyperspectral imaging can acquire a large number of samples in the same time as well as a large amount of spectral data. Machine learning method is the key to analyzing the spectral information and exploring the relationship between spectral information and the predicted features. Various discriminant methods used for classification of seed varieties have showed good results, such as linear discriminant analysis (LDA) [14,15], partial least-squares discriminant analysis (PLS-DA) [16,17], support vector machine (SVM) [18], and artificial neural networks (ANN) [15], etc.

Nowadays, as an emerging machine learning method, deep learning has gain great attentions from different fields [19,20,21]. Deep learning method learns data feature automatically and deeply, which enables it to perform well with large volume of data. Deep learning is mainly used to analyze 2D images [22], and researchers have also extended it to the processing of 3D images [23] and 1D signal [24]. Deep learning for hyperspectral image analysis is primarily developed for remote sensing [25]. Recently, deep learning has been applied to vibrational spectral data analysis for classification [26] and regression [27].

The object of this study was to identify cotton seed varieties using near-infrared hyperspectral imaging with deep learning. The specific objectives were: (1) Explore the differences among different varieties of cotton seeds based on spectral information; (2) Develop deep learning architectures for cotton seed variety identification; (3) Build PLS-DA, SVM and LR models used for comparison.

## 2. Results and Discussion

### 2.1. Spectral Profiles of Cotton Seeds

**Figure 1:**
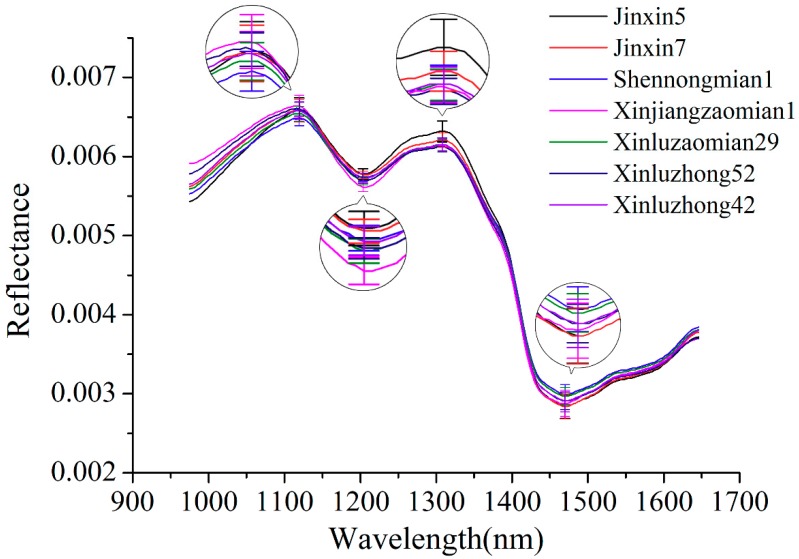
Average spectra of seven varieties of cotton seeds with standard deviation of four wavelengths (peaks: 1119 and 1308 nm; valleys: 1204 and 1470 nm).

Figure 1 shows the average spectral reflectance curves of seven varieties of cotton seeds. Spectra of all cotton seeds of each variety were averaged to obtain average spectrum of the corresponding variety. The standard deviation (SD) of four typical wavelengths (peaks: 1119 and 1308 nm; valleys: 1204 and 1470 nm) were also presented. The waveband at 1119, 1204 and 1308 nm might related to the second overtone of C–H stretch [28]. The spectral wavelength at 1470 nm can be attributed to the O–H stretch first overtone [29]. It can be seen that similarities could be clearly observed among the change tendency of seven spectral reflectance curves. Although some differences could be found for the seven varieties of cotton seeds, the differences are distributed over several wavebands. Overlaps could also be found. Thus, further analyses should be conducted to discriminate different varieties of cotton seeds.

### 2.2. Analysis of PCA Score Images

**Figure 2:**
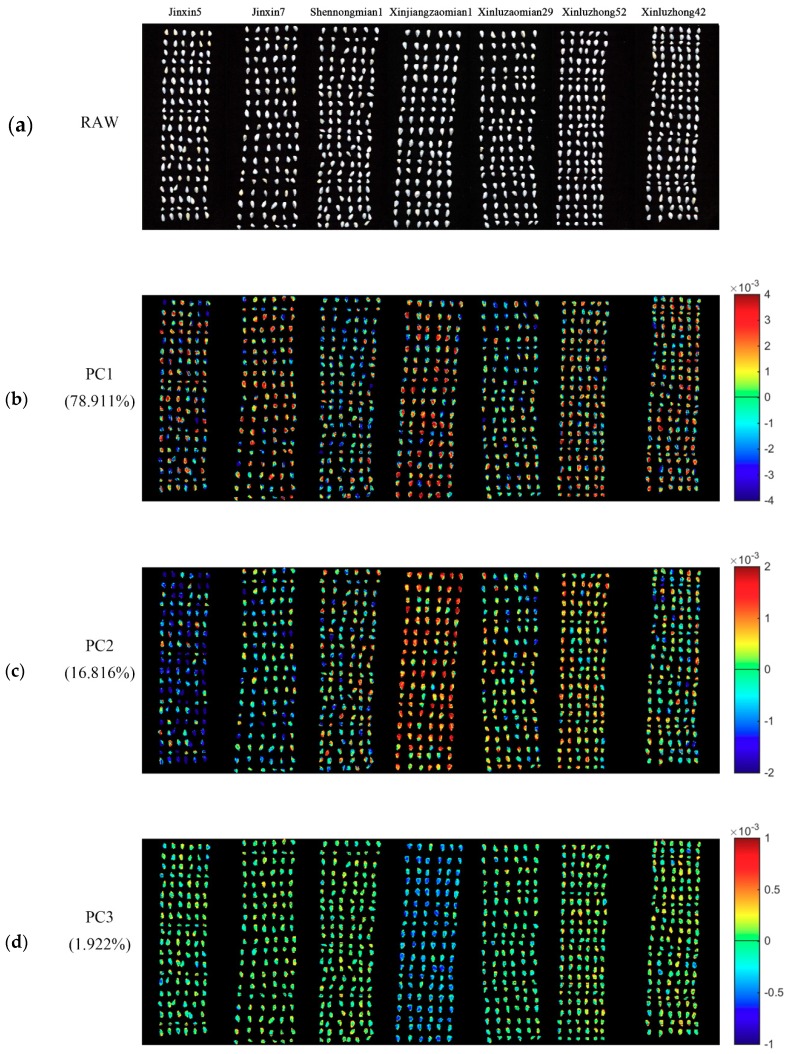

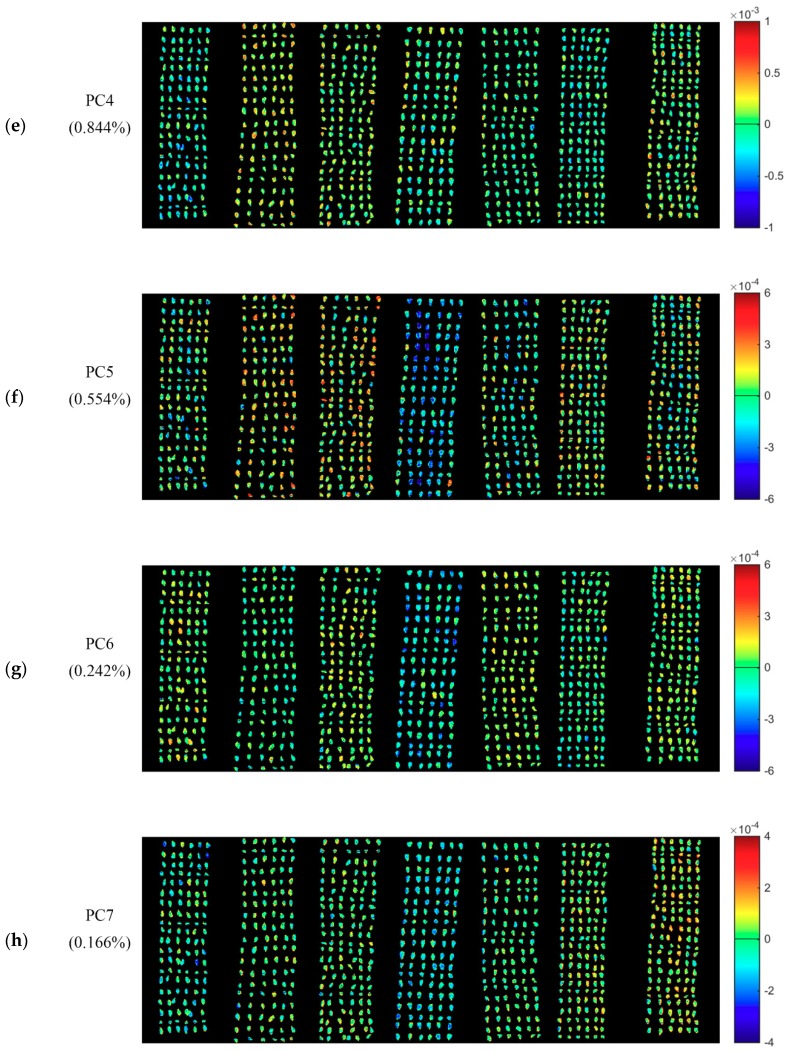

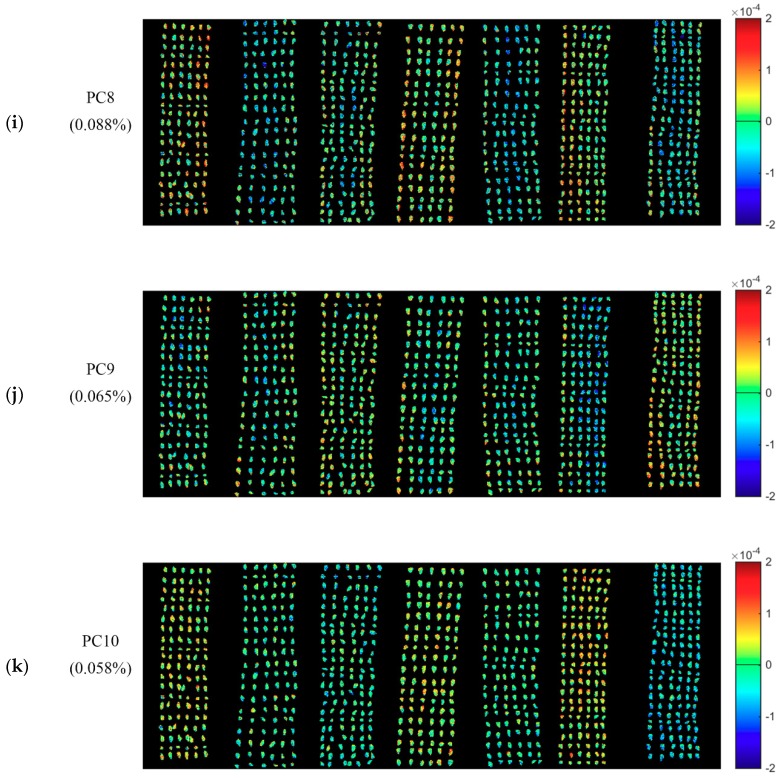
Pseudo raw image of the seven varieties of cotton seeds and the PCA score images of the first ten PCs. The letter (**a**) represent the pseudo raw image (1000, 1200 and 1400 nm); (**b**–**k**) represent the PCA score images of PC1–PC10. Numbers in the brackets are percentage of explained total variance.

PCA was conducted for the qualitative analysis. One hyperspectral image of each variety was randomly selected. PCA was conducted on pixel-wise spectra within the seeds in the hyperspectral images of seven varieties of cotton seeds. Each pixel had score values of different PCs. Knowing the scores value of each pixel of a certain PC, it was easy to obtain the pixel-wise PCA score image (Figure 2). Since the first ten PCs (99.667%) contain most of the information of cotton seeds, PCA score images of PC1-PC10 were obtained to show commonalities and differences among different cotton seeds. As shown in Figure 2, Figure 2b,k show the PCA score images of PC1-PC10, respectively, and the numbers in the left side represent the explained information of each PC.

Different varieties of cotton seeds could be divided into different groups according to color distribution within seed kernels. As well, different division could be found in PCA score images of different PCs.

For PCA score image of PC1, the color distribution of cotton seeds could be divided into three groups (Group 1: Category 0, category 1 and category 3; Group 2: category 2 and category 4; Group 3: category 5 and category 6). Group 1 is primarily in yellow and red color, and Group 2 is mainly in green and blue colors. However, there is no clear color bias in Group 3. For PCA score image of PC2, colors of several varieties of cotton seed show obvious differences. Most parts of category 0 is dark blue, and category 3 is primarily composed of red and orange colors, while category 5 is overall in yellow tones.

Although PC3-PC10 explained less information, some differences revealed by color distribution could still be found among samples. For PCA score images of PC3, PC5, PC6 and PC7, category 3 contains more blue areas, which is the most obvious difference with the other six varieties. Thus, there are two groups (Group 1: Category 3; Group 2: the other 6 categories). Three groups (Group 1: Category 0; Group 2: category 1; Group 3: The other 5 categories) of color distribution could be found in PCA score image of PC4: Category 0 is mainly in light blue color; and category 1 is most in orange and yellow tones, while seeds of the rest categories are mixed blue with yellow. For PCA score images of PC8, there are two groups (Group 1: Category 0, category 3 and category 5; Group 2: category 1, category 2, category 4 and category 6) of color distribution. For PCA score images of PC9, there are three groups (Group 1: Category 0-category 4; Group 2: category 5; Group 3: category 6) of color distribution. Category 6 could be clearly distinguished with mainly blue color in PCA score image of PC10.

Category 1, 2 and 4 show slight differences with other varieties in the PCA score images of PC1-PC10, indicating further classification using discrimination models were needed.

### 2.3. Effective Wavelength Selection

The existence of uninformative variables might influence the modeling speed and accuracy. Thus, it is necessary to select effective wavelengths contributing more to the classification in order to reduce the data volume. As shown in PCA score images of the first ten PCs, differences among the seven varieties of cotton could be observed in each PC score image, and these PCs explained 99.667% of total variance. Thus, loadings of the ten PCs were used for effective wavelength selection.

**Figure 3:**
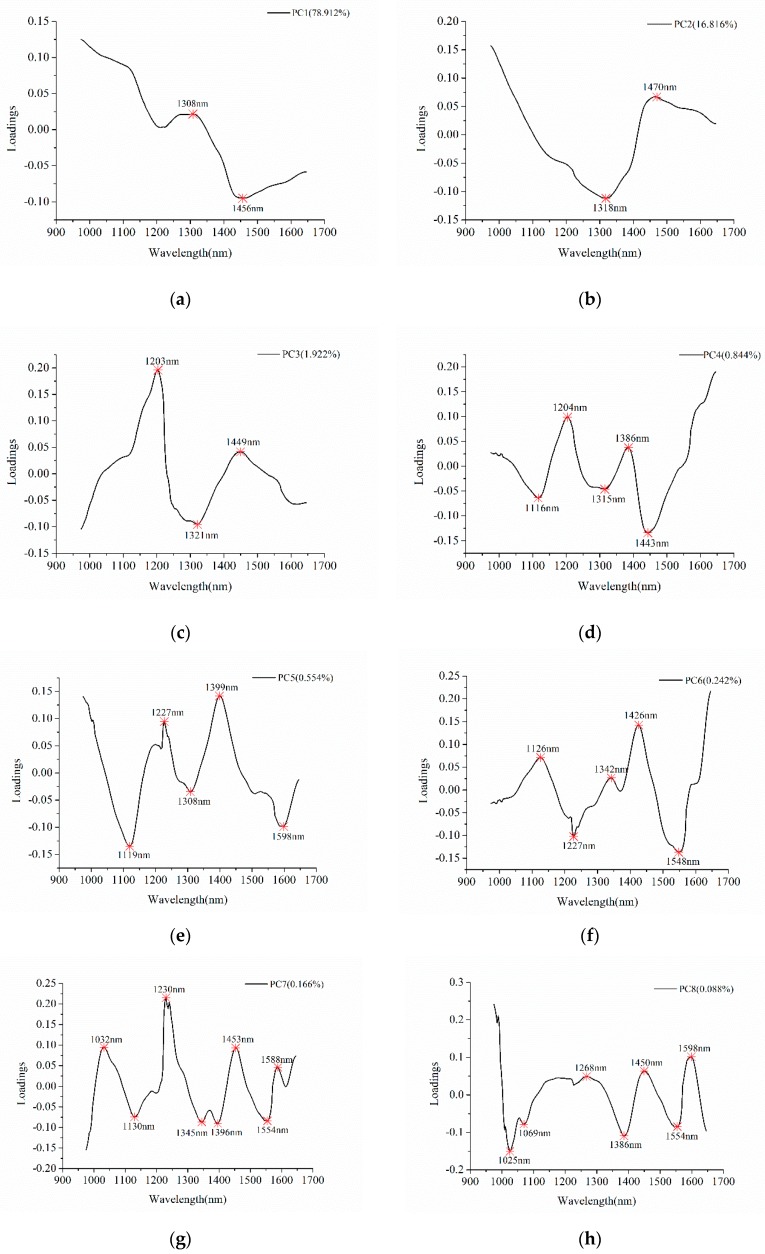

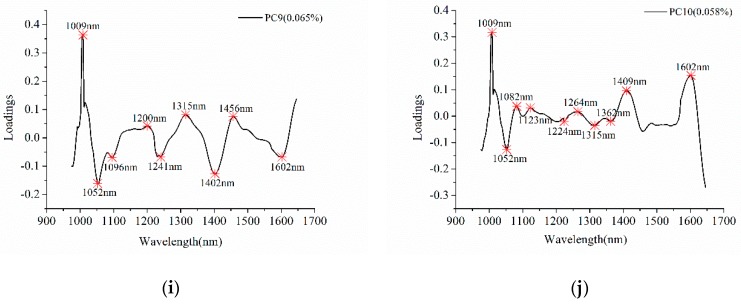
Effective wavelengths selection using the first ten PCs. The letters from (**a**–**j**) represent the PCs from PC1 to PC 10.

Figure 3 shows the detail of effective wavelengths selection. Table 1 summarized the selected effective wavelengths according to the loadings of PC1–PC10, and a total of 43 effective wavelengths were chosen for further analyses. Comparing with full spectra, the number of variables for effective wavelengths is reduced by 78.5%. As shown in Table 1 and Figure 3, some of the wavelengths were selected as effective wavelengths according to loadings of different PCs, and some successive wavelengths were selected.

### 2.4. Discriminant Models using Full Spectra and Effective Wavelengths

Two different CNN architectures were used to classify cotton seed varieties, including a self-design CNN architecture and a ResNet architecture. As mentioned above, the two CNN models used SoftMax function as classifier. For comparison, LR, PLS-DA and SVM models, which were widely used discriminant models in spectral data analysis, were built using full spectra or effective wavelengths. In addition, LR, PLS-DA and SVM which is widely used in traditional CNN models were also used to replace SoftMax function. They are used to handle the classification tasks based on the features extracted by the two CNN architectures for further improvement. Table 2 shows the results of different classification models using full spectra or effective wavelengths for cotton seed varieties identification.

For models using full spectra, classification results were decent. Most of models obtained the classification accuracy over 80%. Among eleven models using full spectra, PLS-DA model obtained the worst results, with the accuracy of calibration (81.764%), validation (79.947%) and prediction set (80.401%) all lower than other models. CNN-SVM and SVM models obtained similar results, with accuracy around 93%, 89% and 88% for calibration, validation and prediction sets, respectively, while CNN-SVM obtained slightly better performances in calibration and validation sets than SVM model. Except SVM model, pure LR and PLS-DA models performed worse than the corresponding LR and PLS-DA models combined with deep learning. For LR and PLS-DA models based on the CNN and the ResNet architecture, the accuracy was improved by about 10%, 14% for calibration set, respectively. The reason could be attributed to the strong feature learning ability of deep learning. Compared with the self-design CNN based models, ResNet based models performed better in the calibration set. However, for the validation and prediction sets, self-design CNN based models achieved better results, with accuracy in the range of 86–89%.

For classification models using effective wavelengths, LR model obtained the worst results, with classification accuracy of calibration, validation and prediction sets all lower than 70%. Compared with LR models, CNN-SVM and SVM obtained better performances, with classification accuracy of calibration set over 89% and classification accuracy of validation and prediction sets over 84%. The self-design CNN based models achieved classification accuracy of calibration, validation and prediction all over 80%. ResNet based models showed better performances for calibration set with classification accuracy over 90%, but classification accuracy of validation and prediction was lower than 80%. In summary, the self-design CNN based models performed worse in calibration set but obtained better performance in validation and prediction sets than ResNet based models.

The classification models using full spectra performed better than those using effective wavelengths. LR model using full spectra performed significantly better than LR model using effective wavelengths. The self-design CNN based models and ResNet based models using full spectra also performed better than those using effective wavelengths. The reason was that full spectra contained more information than the selected effective wavelengths. What’s more, dealing with the simple data sets by deep learning couldn’t fully reflect the advantage of deep learning. The differences of performances of self-design CNN based models and ResNet based models indicating that CNN architectures had influence on classification performances. Performances of deep learning models varied due to the different classifiers, indicating the influence of classifiers on model performances.

**Figure 4:**
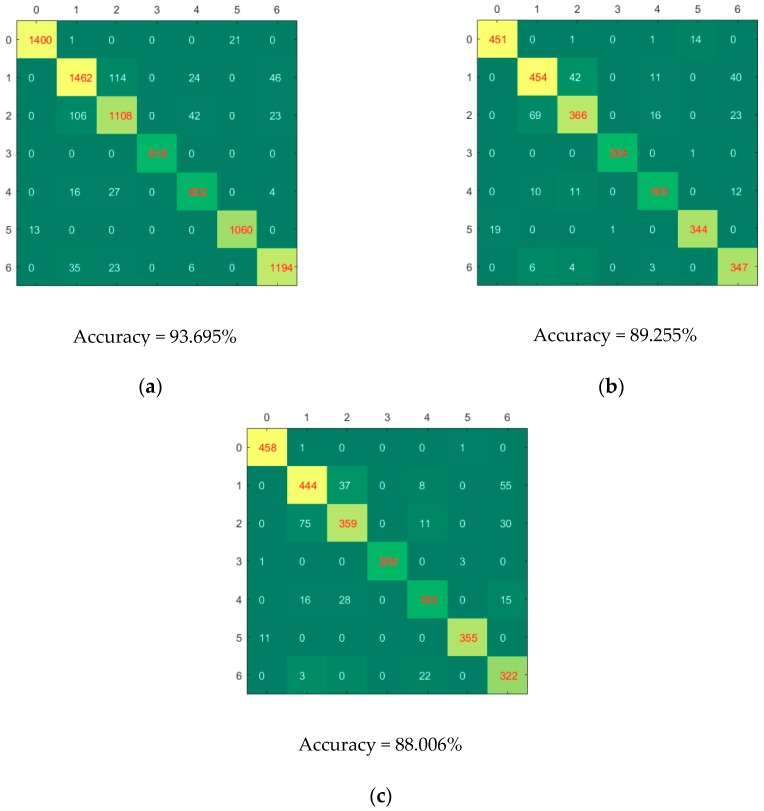
The confusion matrices of calibration (**a**), validation (**b**) and prediction (**c**) for CNN-SVM model using full spectra.

Figure 4 shows the confusion matrices of calibration, validation and prediction for CNN-SVM model using full spectra. Good classification performances could be found for category 3 (Xinjiangzaomian1) and category 5 (Xinluzhong52), for which few samples were misclassified. Category 0 (Jinxin5) were more likely to be misclassified as category 5. Category 1 (Jinxin7) were more likely to be misclassified as category 2 (Shennongmian1), category 4 (Xinluzaomian29) and category 6 (Xinluzhong42). Category 4 was more likely to be misclassified as category 1, category 2 and category 6. Category 6 was more likely to be misclassified as category 1, category 2 and category 4. Most of the cotton seeds could be accurately classified, which indicated that as a rapidly and non-destructively method, hyperspectral imaging coupled with deep learning could be used to identify cotton seed varieties.

## 3. Materials and Methods

### 3.1. Sample Preparation

Seven different varieties of cotton seeds were collected in 2016 from Shihezi, Xinjiang Uyghur Autonomous Region, China. These seven varieties were Jinxin5, Jinxi7, Shennongmian1, Xinjiangzaomian1, Xinluzaomian29, Xinluzhong52 and Xinluzhong42, and the corresponding number of seeds used in the study were 2353, 2497, 2242, 1031, 1122, 1804 and 2111, respectively. All these seeds were sound. The sulfuric acid solution was used for the depilation treatment of cotton seeds. The H_2_SO_4_ solution (75% mass fraction) heated to 100 °C was slowly pour into a ceramic container with cotton seeds placed inside until the cotton seeds were submerged, and then the seeds were rapidly stirred until the fluff on the seeds was completely removed. Finally, the cotton seeds were rinsed with water until the water was clear, and then the cotton seeds were air-dried before the acquisition of hyperspectral image. For further analysis, category values of the seven varieties of cotton were assigned as 0, 1, 2, 3, 4, 5, 6 (corresponding to Jinxin5, Jinxi7, Shennongmian1, Xinjiangzaomian1, Xinluzaomian29, Xinluzhong52 and Xinluzhong42, respectively). To build calibration models, the cotton seeds were randomly divided into the calibration set, the validation set and the prediction set at the ratio of 3:1:1.

### 3.2. Hyperspectral Image Acquisition and Correction

A line-scan near-infrared hyperspectral imaging system with the spectral range of 942–1646 nm was used to acquire hyperspectral images of cotton seeds. The system contains an imaging spectrograph (ImSpector N17E, Spectral Imaging Ltd., Oulu, Finland) integrated with a Xeva 992 camera (Xenics Infrared Solutions, Leuven, Belgium) which uses an OLES22 lens (Spectral Imaging Ltd., Oulu, Finland). The light source is two 150W tungsten halogen lamps (3900 Lightsource, Illumination Technologies Inc., Elbridge, NY, USA). The line scan is conducted by moving the sample plate using a stepper motor (Isuzu Optics Corp., Taiwan, China).

To acquire hyperspectral images, the system parameters were firstly adjusted to acquire clear and non-deformable images. The camera exposure time, the sample plate moving speed and the distance between sample plate and the camera were set as 3 ms, 11.5 mm/s and 14 cm. Then white reference image and dark reference image were acquired using the adjusted system. The white reference image was acquired using a piece of pure white Teflon board with nearly 100% reflectance, and the dark reference image was acquired by turning off the light source and covered the camera lens with lens cap.

To acquire hyperspectral images, single cotton seeds were placed separately. In all, the number of hyperspectral images of Jinxin5, Jinxin7, Shennongmian1, Xinjiangzaomian1, Xinluzaomian29, Xinluzhong52 and Xinluzhong42 were 19, 20, 17, 8, 9, 14 and 16.

After image acquisition, the images were corrected to calibrate light intensity and reduce dark current. The image correction was conducted using the equation:

(1) $$I_{c}=\frac{I_{\mathrm{raw}}-I_{\mathrm{dark}}}{I_{\mathrm{white}}-I_{\mathrm{dark}}}$$

where *I*_c_ is the corrected image, *I*_raw_ is the raw image, *I*_white_ is the white reference image and *I*_dark_ is the dark reference image.

### 3.3. Spectral Data Preprocessing and Extraction

To extract spectral data, each cotton seed was defined as the region of interest. Cotton seeds were firstly isolated from the background. A binary image was formed by the gray-scale image at 1200 nm, in which seed regions were ‘1′ and the background regions were ‘0′. The binary image was then applied to the gray-scale images at each wavelength to remove the background. The outer ring of each cotton was eliminated, and pixel-wise spectra were firstly preprocessed by wavelet transform (wavelet function Daubechies 10 with decomposition level 3) followed with an area normalization. Then average spectrum was calculated from pixel-wise spectra within each cotton seed to represent the seed.

### 3.4. Multivariate Analysis

#### 3.4.1. Principal Component Analysis

Principal component analysis (PCA) is a widely used qualitative analysis and data reduction method which could be used to explore data features. PCA conducts linear transformation to transform the original variables into a set of new orthogonal variables. These new variables were ranked according to the variance. The first variable with the largest variance was the first principal component (PC1), and the rest are defined in the same manner. The first few PCs contain the most useful information, and generally the first few PCs are used for qualitative analyses and data reduction. In hyperspectral images, each pixel has a spectrum. PCA can be conducted on pixel-wise spectra, and scores of each pixel at each PC can be obtained. The pixel-wise score values of each PC can be presented by color gradients, which is the PCA score images. Differences among samples can be observed intuitively from the PCA score images [30].

#### 3.4.2. Partial Least Squares Discriminant Analysis

Partial least squares discriminant analysis (PLS-DA) is the most widely used supervised discrimination method. PLS-DA is performed in the same way as partial least squares regression (PLSR). PLS-DA uses a set of dummy numbers referring to the sample categories as the dependent variables, and then PLS-DA conducts the same procedures as PLSR to explore the linear relationship between the independent variables and the dependent variables [31]. Specially, the categories of the seven varieties of cottons for PLS-DA were assigned as 0000001, 0000010, 0000100, 0001000, 0010000, 0100000 and 1000000 (corresponding to Jinxin5, Jinxi7, Shennongmian1, Xinjiangzaomian1, Xinluzaomian29, Xinluzhong52 and Xinluzhong42, respectively).

#### 3.4.3. Logistic Regression

Logistic regression (LR) is a linear model for classification, which can fit binary or multinomial logistic regression [32]. On the basis of linear regression algorithm, a sigmoid function is added to map the linear combination of independent variables into the value in the range [0,1]. LR algorithm can be applied with optional *L1*, *L2* regularization. It also can be extended to multi-class classification tasks using One-vs-Rest.

#### 3.4.4. Support Vector Machine

Support vector machine (SVM) is a widely used pattern recognition algorithm. SVM aims to construct a hyperplane or a set of hyperplanes to separate the samples from different classes maximally. For samples which cannot be linearly classified, kernel functions are applied to map the original data into high-dimension space. The new data in the high-dimension space may be linearly separable. Radial bias function (RBF) is a widely used kernel function, and it can deal with linear and non-linear issues effectively. In this study, SVM with RBF kernel function was used to establish discriminant models. For PLS-DA models, leave-one-out cross validation were used. For SVM models, a five-fold cross validation was used. A grid-search procedure was used to find the optimal parameters of SVM models, i.e., the penalty coefficient (C) and the kernel parameter (γ) [33].

#### 3.4.5. Deep Learning Methods

Deep learning algorithms have become a kind of useful method for spectra data analyses in agriculture [21]. Convolutional neural network (CNN) is one of the well-known deep learning structures for feature extraction, classification and regression. In this study, two kinds of one-dimension CNN [26] architectures were designed and evaluated to achieve feature representation from input spectra data and realize classification based on extracted deep features.

**Figure 5:**
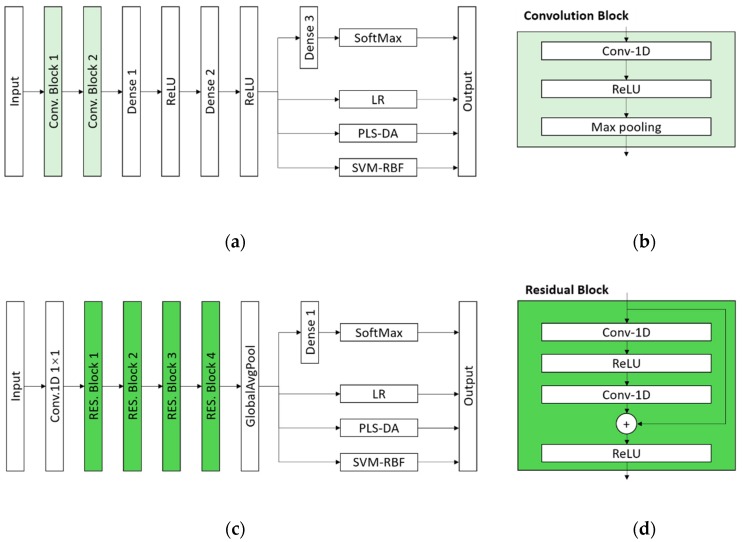
The architectures of proposed classification models: (**a**) The architecture of CNN-SoftMax, CNN-LR, CNN-PLS-DA and CNN-SVM; (**b**) the architecture of the Convolution Block; (**c**) the architecture of ResNet-SoftMax, ResNet-LR, ResNet-PLS-DA and ResNet-SVM; (**d**) the architecture of the Residual Block.

The first architecture is shown in Figure 5a, which consists of two convolution blocks (Conv. Block), a fully connected network with three dense layers, and a SoftMax layer. The function of SoftMax layer is as follows [34]:

(2) $$\sigma {\left(z\right)}_{i}=\frac{e^{z_{i}}}{\sum_{k=1}^{K}e^{z_{k}}}$$

where *K* is the number of elements of the input vector, *z_i_* is the *i*th element of the input vector and *σ*(*z*)_*i*_ is the *i*th element of the output vector.

The Conv. Block is built by a one-dimension convolutional layer with a ReLU activation and a max pooling layer, which is described in Figure 5b. The numbers of kernels are 64 and 128 for Conv-1D layers in Conv. Block 1 and Conv. Block 2, respectively. The numbers of the neurons in Dense 1, Denses 2 and 3 were defined as 512, 128, 7, respectively. The second architecture is a one-dimension-convolution based ResNet (ResNet-1D) architecture shown in Figure 5c, including a 1 by 1 convolution layer, four residual blocks (RES. Block), a global average pooling (GlobalAvgPool) layer, a dense layer with 7 neurons and a SoftMax layer. The design of ResNet-1D structure refers to the well-known ResNet for two-dimension image classification [35]. The detail information of the RES. Block is shown in Figure 5d. The numbers of kernels in two Conv-1D layers are same in each RES. Block, which were defined in turn as 64, 128, 256, 512.

All convolutional layers in the two mentioned CNN models used a kernel size of 3, stride of 1. The training procedure for both of two mentioned CNN models was carried out to minimize the SoftMax Cross Entropy Loss using Stochastic Gradient Descent (SGD).

Considering that SoftMax is not the best classifier, we used LR, PLS-DA, SVM as classifier for comparison in Figure 5. The activated output of Dense 2 layer in Figure 5a and the output of GlobalAvgPool layer in Figure 5c were defined as the input value for LR, PLS-DA and SVM models.

#### 3.4.6. Effective Wavelength Selection

Spectral data extracted from the hyperspectral images contains redundant and uninformative information. Selection of informative variables contributing more to prediction is of great importance. Variable selection can reduce the number of input variables to simplify models and improve the modelling efficiency. In this study, loadings of PCA were used for effective wavelength selection [36].

Loadings are regression coefficients between original variables and the corresponding PCs, which could be obtained during the linear transformation of PCA. The absolute loading value indicates the importance of the corresponding variables. The larger the absolute loading value, the more important the variable is. Therefore, variables with larger absolute loading value of each PC can be selected as effective wavelengths.

#### 3.4.7. Model Evaluation and Software

The performances of models were evaluated by the classification accuracy, which was defined as the ratio of the number of correctly classified seeds to the total number of cotton seeds. PLS-DA and SVM were conducted on Matlab R2014b (The Math Works, Natick, MA, USA). LR model was programmed using scikit-learn machine learning package in Python 3. Deep learning models were conducted on Python 3 and MXNET framework (Amazon, Seattle, WA, USA).

## 4. Conclusions

Hyperspectral imaging coupled with deep learning was successfully used to identify cotton seed varieties. PCA score images of the first ten PCs illustrated the differences among the seven cotton seed varieties. A total of 43 effective wavelengths were selected by loadings of the first 10 PCs. Two different CNN architectures, including a self-design CNN model and a ResNet model, using SoftMax function, PLS-DA, LR and SVM as classifiers all obtained good performances based on full spectra or effective wavelengths. Pure PLS-DA, LR and SVM models were also built using full spectra or effective wavelengths. CNN-LR and CNN-PLS-DA models performed better than pure LR and PLS-DA models. Variations on classification performances of self-design CNN and ResNet based models showed the influence of classifiers in deep learning models. The different performances between self-design CNN based models and ResNet based models illustrated the influence of CNN architectures. Classification models using full spectra performed better than those using effective wavelengths, and the differences were larger for deep learning based models. The overall result illustrated that deep learning models could be applied to identify cotton seed varieties. In future studies, sample number and different deep learning architectures should be taken into consideration to improve the accuracy and robustness of classification model.

## Figures and Tables

**Table 1:** Effective wavenumbers selected by PCA loadings.

Methods	No.	Effective Wavelengths (nm)
PCA loadings	43	1009, 1025, 1032, 1052, 1069, 1082, 1096, 1116, 1119, 1123, 1126, 1130, 1200, 1204, 1224, 1227, 1230, 1241, 1264, 1268, 1308, 1315, 1318, 1321, 1342, 1345, 1362, 1386, 1396, 1399, 1402, 1409, 1426, 1443, 1450, 1453, 1456, 1470, 1548, 1554, 1588, 1598, 1602

**Table 2:** Results of classification models using full spectra and effective wavelengths.

Classifier	Full Spectra (%)			Effective Wavelengths (%)		
	Calibration	Validation	Prediction	Calibration	Validation	Prediction
CNN-SoftMax ^a^	91.191	89.065	88.838	87.629	84.071	82.860
CNN-LR	94.060	88.611	87.752	90.070	83.731	83.276
CNN-PLS-DA	91.112	88.082	86.644	87.088	82.709	82.027
CNN-SVM	93.695	89.255	88.006	89.970	84.487	84.260
ResNet-SoftMax	95.381	85.698	86.039	92.273	79.985	79.228
ResNet-LR	99.585	84.335	82.324	98.238	76.040	75.952
ResNet-PLS-DA	95.130	85.585	85.358	91.707	78.509	77.677
ResNet-SVM	96.325	85.963	85.887	94.098	79.153	79.115
LR	84.156	82.406	83.012	62.736	62.429	65.305
PLS-DA	81.764	79.947	80.401	78.870	77.261	77.147
SVM	93.557	89.217	88.422	89.441	84.147	84.033

^a.^ CNN-SoftMax means using SoftMax function as classifier for the CNN model.
